# Supplementary material for: Epstein–Barr Virus Infection of Pseudostratified Nasopharyngeal Epithelium Disrupts Epithelial Integrity
Source: Cancers (Basel). 2020 Sep 22;12(9):2722. doi: 10.3390/cancers12092722 (PMC7564236; doi:10.3390/cancers12092722)
Supplement: Supplementary file 1 [file cancers-12-02722-s001.zip › cancers-889431 supplementary Figure 1.pdf]

Supplementary figure 1

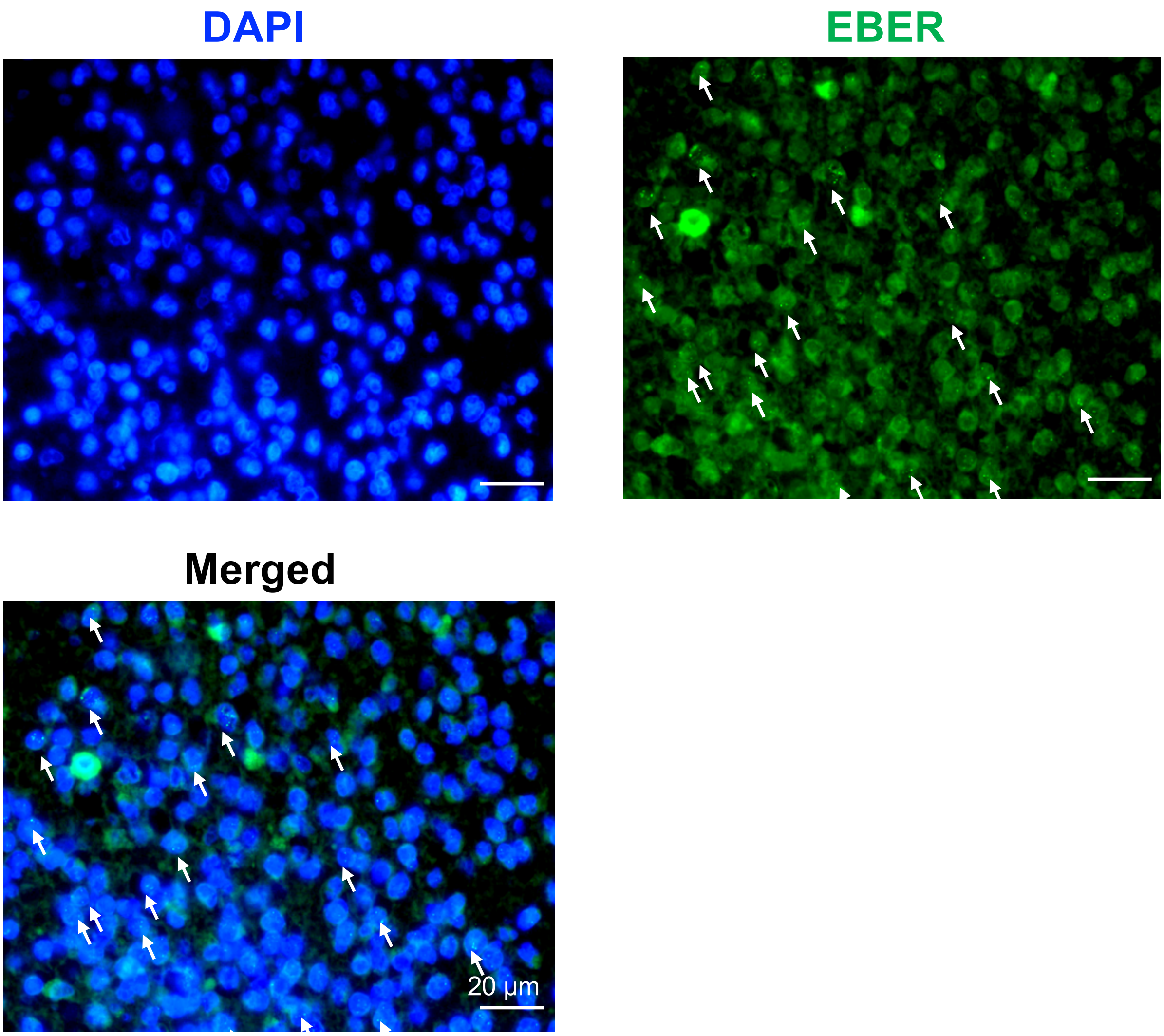

Figure S1. EBER1 staining of EBV-positive NPC tumor tissue: White arrows indicate cells showing EBER1-positive green puncta signals in the nuclei of tumor cells. (left top) Nuclei are stained blue by DAPI. (right top) EBER1 *in situ* hybridization (ISH) signals of strong and discrete puncta in the nuclei are shown in green. (left bottom) Merged picture of EBER1 signals and DAPI staining is shown.
